# Psychological Distress and Interventions for Older Victims of Crime: A Systematic Review

**DOI:** 10.1177/15248380221130354

**Published:** 2022-11-03

**Authors:** Jessica Satchell, Tabitha Craston, Vari M. Drennan, Jo Billings, Marc Serfaty

**Affiliations:** 1 University College London, London, UK; 2 Kingston University, Surrey, UK

**Keywords:** crime victims, older adults, psychological distress, psychological interventions, systematic review

## Abstract

We aimed to conduct the first systematic narrative review and quality appraisal of existing evidence on the psychological consequences of crime in older victims in the community and psychological interventions. We searched five databases to identify all peer-reviewed literature published in English on psychological impact and/or interventions for older crime victims and quality appraised these using the Mixed-Methods Appraisal Tool, following Preferred Reporting Items for Systematic Reviews and Meta-analyses guidelines (Prospero: CRD42019140137). Evidence from included studies were narratively synthesized, along with their strengths and limitations. We found 20 studies on psychological distress in older victims, four of which included interventions. From these, we identified 30 different impacts including symptoms of anxiety, depression, post-traumatic stress disorder, emotions including humiliation and self-blame, and behavioral changes. Only feasibility interventions have been published, although promising results were reported for cognitive-behavioral informed treatments for depression and anxiety. Studies were wide-ranging in aims, crimes included, and outcomes used. Recommendations for improving the evidence-base and to raise the profile of this neglected population have been provided.

## Introduction

Supporting older people after a crime is a growing challenge with global population ageing and is an issue of social concern internationally (Burnes et al., 2017; HMICFRS, 2019; Muhammad et al., 2021; Qin & Yan, 2018). Even in cities considered comparatively safe such as London, an estimated 34,133 adults aged 65 years or over reported a crime in 2019 (Metropolitan Police Service, personal communication, 2019; The Economist, 2022), and as 60% of crimes go unreported, the true figure is likely to be even higher (MacDonald, 2002). Older adults are also at greater risk of specific crimes including fraud and distraction burglary (Laycock, 2020; Qin & Yan, 2018). In the United States, an estimated 1 in 18 older adults in the community suffered fraud each year before Covid-19 (Burnes et al., 2017), and as fraud reports significantly increased during the pandemic, even greater numbers are now likely to have been affected (Office for National Statistics, 2022; Payne, 2020). Internationally, theft and assault are reported to have resurged as lockdown restrictions have eased and social movement resumed (Langton et al., 2021; Nivette et al., 2021) and data on rates of different crimes during Covid-19 are still emerging. With costs of living rising in many countries, there are growing concerns that crime will continue to increase (International Monetary Fund, 2022; Mayor of London, 2022). Globally, the number of older crime victims is likely to be significant.

There is growing recognition of the impact of crime as a public health concern (Burnes et al., 2017; Tan & Haining, 2016) Historically, public health and criminology developed independently of one another meaning the links between crime victimization and health outcomes are poorly understood (Tan & Haining, 2016). Older adults in the community have been further neglected from research on crime and health due to an enduring and disproportionate focus on explaining the “Victim’s Paradox”: the perception that older adults are more fearful of crime than younger age groups despite being at statistically less risk overall (Lindquist & Duke, 1982). This overlooks that large numbers of older adults are still affected and that they are at increased risk of specific crimes (Laycock, 2020). It also implies that fear of crime in older adults is irrational yet impact may be greater in this group because of concurrent life events including declining physical health, bereavement, and reduced income in retirement (Jackson, 2009). Severe and prolonged symptoms of psychological distress have been identified in older victims of all crime types (Serfaty et al., 2016). Burglary and interpersonal violence in older adults has also been associated with accelerated mortality and increased risk of nursing home placement (Donaldson, 2003; Lachs et al., 2006).

Recent reviews have furthered understanding of the psychological impact of domestic violence and elder abuse in older people (e.g., Knight & Hester, 2016; Yunus et al., 2019) but the literature on other crime types has not been synthesized. Domestic and elder abuse should be studied separately as they are typically ongoing and committed by someone in a close existing relationship or with an expectation of trust (WHO, 2017). This is only one aspect of crime which affects older people. For example, ten years of law enforcement data suggest that almost half (43%) of violent victimizations in older people are committed by strangers (Langton & Truman, 2014). Understanding the unique psychological needs and how to support older victims of other crime types, such as burglary, theft, criminal damage, stranger assault, and fraud, is also important.

### Study Aims

We aimed to address the following questions: (a) What is the psychological impact of crime in older victims? (b) What psychological interventions are there for distress in older crime victims? (c) What are the strengths and weaknesses of the existing literature and how can the evidence base be improved?

## Methods

### Study Protocol

Our review adheres to guidance from Cochrane and the Preferred Reporting Items for Systematic Reviews and Meta-analyses (PRISMA) (Higgins et al., 2021; Page et al., 2021). We registered the review protocol on PROSPERO on August 14, 2019 (CRD: 42019140137) and submitted amendments on February 28, 2022, and May 12, 2022.

### Inclusion and Exclusion Criteria

We included peer-reviewed studies of any design published in English from 1980 to 2022 that presented data on psychological impact or psychological interventions for community-dwelling older crime victims aged 50 years and over. Psychological impact was defined as any emotional or behavioral response (Ridner, 2004), which could be recorded objectively or subjectively. Psychological interventions could be any format and include any comparator, including treatment-as-usual and no care. As there is no agreed definition of “older adults,” we selected a conservative definition of age 50 years and over, as this was lowest age identified in a review of ageing definitions, and it is recommended that reviews be over-inclusive to capture all relevant literature (Cosco et al., 2014).

We excluded studies on crimes perpetrated by friends, family, or caregivers, unless analyzed and reported separately, as psychological impact may be different when the victim is in a close-existing relationship with the perpetrator (Langton & Truman, 2014). Reviews on psychological impact in older victims of domestic violence and carer abuse have also recently been published elsewhere (Knight & Hester, 2016; Yunus et al., 2019). Studies on dementia, serious mental illness, personality disorder, and intellectual disability were also excluded as the focus is on psychological outcomes rather than preexisting conditions. Studies on alcohol or substance misuse were excluded as these may be considered a consequence of distress rather than a direct outcome.

### Search Strategy

We used PsycINFO, MEDLINE, Embase, Cumulative Index to Nursing and Allied Health Literature, and PTSDPubs to search existing literature published between 1980 and August 2019. We updated the search in August 2021 and April 2022. A broad date range of over four decades was chosen to identify as much relevant literature as possible. The key words were “older adult” AND “mental health” AND “crime victim.”

Two reviewers independently screened titles and abstracts of all search results and assessed the full-text of potentially relevant papers before conferring (99.8% agreement, *k* = 0.66, 95% confidence interval [CI] [0.52–0.79], “substantial agreement”; Cohen, 1960). A third reviewer was nominated to make final decisions on papers where eligibility remained unclear even after discussion. Reference lists of all included papers were also screened. The Cochrane Central Register of Controlled Trials and International Standard Randomized Controlled Trial Number registry were also searched.

### Data Extraction and Quality Appraisal

The following data were extracted into pre-populated tables: study design, setting, sample size, sample characteristics, crimes included, mental health outcomes, recruitment procedures, duration between crime occurring and assessment, analytical strategy, lengths, and procedures. Additional data extraction for intervention studies included treatment and comparator characteristics. Missing data was recorded as “not reported.” A second reviewer crosschecked 20% of data extraction and found no errors. Studies were quality appraised using the Mixed Methods Appraisal Tool (MMAT) version 18 (Hong et al., 2018). Low methodological quality was not an exclusion criterion as the review aimed to provide an overview and quality appraisal of existing literature.

### Synthesis

The results were summarized using narrative synthesis as recommended for systematic reviews that include non-RCT studies (Popay et al., 2006). We considered meta-analysis but deemed it unsuitable due to heterogeneity in study and sample characteristics.

## Results

### Study Selection

The searches combined produced 19,856 results which, once duplicates were removed, left 11,980 results which were screened by two reviewers. Most (*n* *=* 11,575) were excluded based on titles and abstracts, but a full-text review was completed for 56. Of these, 36 were excluded because: (A) the crime was committed by a perpetrator in a close relationship with the victim; (B) the study focused on younger victims; (C) the study was a review or protocol paper that did not present data; (D) the outcome was not psychological, or (E) the intervention was not psychological. One intervention study (Acierno et al., 2004) included older victims of domestic violence but clearly reported that this was the minority of the sample and that no differences were found compared to other crime groups in any analyses. As such, we decided to include this study and a protocol amendment explaining this was submitted to Prospero.

**Figure 1.**
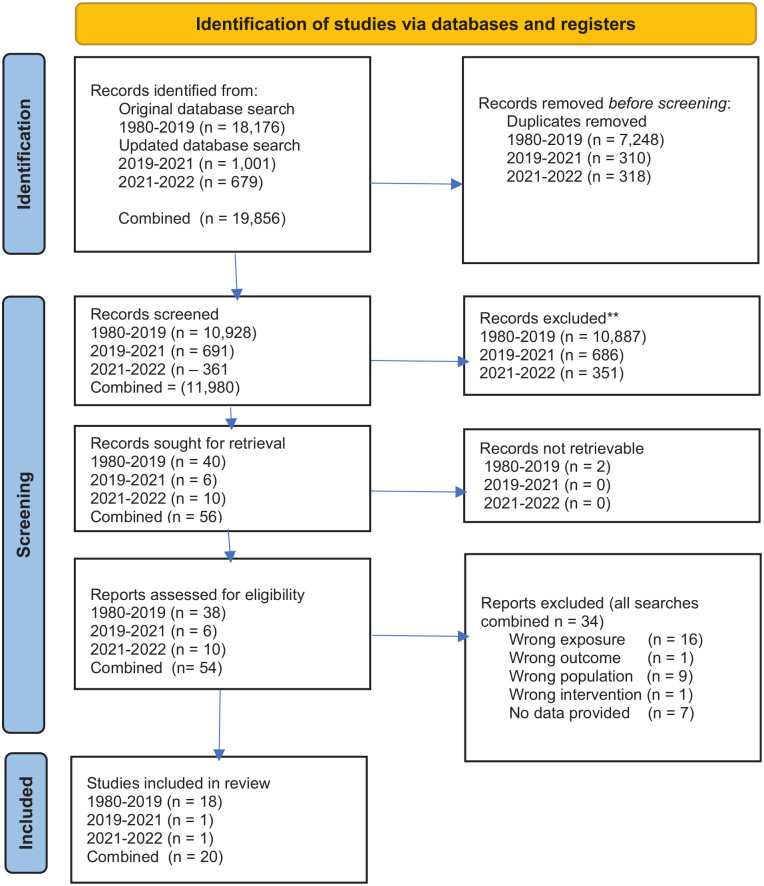
PRISMA 2020 flow diagram for new systematic reviews which included searches of databases and registers only. *Source.* From Page et al. (2021). For more information, visit: http://www.prisma-statement.org/

This resulted in 20 papers for inclusion in this review. Figure 1 illustrates the full selection process using the PRISMA 2020 flow diagram (Page et al., 2021).

### Study Characteristics

Study and sample characteristics of included studies are summarized in Table 1. Of the 20 studies, four included intervention research. The studies were published between 1985 and 2021 and were cross-sectional (*n* *=* 9), case studies (*n* *=* 2), qualitative (*n* *=* 4), randomized controlled trial (*n* *=* 2), descriptive intervention (*n* *=* 1), prospective longitudinal (*n* *=* 1), and secondary analysis (*n* *=* 1). Most (*n* *=* *17*) were from Western countries except for two in India and one in China (Muhammad et al., 2021; Tripathi et al., 2019; Qin & Yan, 2018). The mean sample ages ranged from 60 to 79 years. Crimes included across studies were burglary, vandalism, robbery, mugging, physical assault, sexual assault/ rape, distraction burglary, transphobic hate crime, racial hate crime, fraud, cybercrime, and other scams.

**Table 1.** Summary Characteristics of Studies.

	First Author (Year)	Location	Study Design	Sample Size (*N*)	Mean Age	% Female	% White Ethnicity	Crimes Under Study	Mental Health Outcomes Studied
1	Krause (1986)	USA	Cross-sectional	332	73.4	66%	64%	Crime and legal matters (e.g., robberies, assault, vandalism)	Depressive symptoms
2	Jones (1987)	UK	Case studies	12	Not reported	Not reported	Not reported	Nuisance, vandalism, burglary, fraudulent entry, theft	Describes older victims as upset, distressed
3	O’Neill (1989)	Ireland	Cross-sectional	272	74	Not reported	Not reported	Burglary	Anxiety, depression, sleep disorder, fear of going out, fear of further crime
4	Norris (1992)	USA	Cross-sectional	Not reported	Not reported	Not reported	Not reported	Robbery, physical assault, sexual assault	Post-traumatic stress disorder, perceived stress
5	Tyra (1996)	USA	Case study	1	64	100%	Not reported	Rape	Rape trauma syndrome
6	Simpson (1996)	UK	Cross-sectional	350 + 5	Not reported	Not reported	Not reported	Actual or attempted burglary, robbery, assault, or deceit	Post-traumatic stress disorder
7	Spalek (1999)	UK	Qualitative	25	67	Not reported	Not reported	Pension fraud	Subjectively described anger, anxiety, self-blame,
8	Acierno (2004)	USA	Randomized Controlled Trial	116	66.9	66.50%	53%	Violence	Anxiety, depression
9	McGraw (2006)	UK	Descriptive intervention	77	79	Not reported	Not reported	Distraction burglary	Depression, anxiety
10	Brunet (2013)	France	Prospective longitudinal	39	72.4	64.10%	Not reported	Physical assault	Post-traumatic stress disorder
11	Fredriksen-Goldsen (2014)	USA	Cross-sectional	174	60.97	Transgender sample, no further details provided	79.10%	Transgender victimization (including physical, verbal, or sexual threat or assault, threat of being outed, property damage)	Depressive symptomatology, perceived stress
12	Cross (2015)	Australia	Qualitative	85	Not reported (range 50–83)	45.90%	Not reported	Fraud, attempted fraud	Self-blame, shame, embarrassment
13	Iganksi (2015)	UK	Secondary analysis	Not reported for older adults	Not reported for older adults	Not reported for older adults	Not reported for older adults	Racially motivated hate crime	Internalized emotions (anxiety, panic attacks, crying, tears, depression, difficulty sleeping, fear, loss of confidence, feeling vulnerability, shock) and externalized emotions (anger, annoyance)
14	Tan (2016)	UK	Cross-sectional	280	Not reported	Not reported	Not reported	Violent crimes (threatening/abusive behavior, violent assault, robbery/mugging) and non-violent crimes (burglary, break-in, vehicle-related theft, theft of credit card, other forms of theft, vandalism/property damage)	Stress, sleeping difficulties, lack of confidence, depression, panic attacks
15	Serfaty (2016)	UK	Longitudinal + pilot Randomized Controlled Trial	581 (26 RCT)	71.7	55.20%	86.10%	Burglary, pickpocket, fraud, criminal damage, assault, harassment, theft	Depression, anxiety, post-traumatic stress disorder
16	Reisig (2017)	USA	Cross-sectional	2000	72.42	63.70%	90.40%	Theft, fraud, violence	Depressive symptoms
17	Qin and Yan (2018)	China	Cross-sectional	453	72.29	56.30%	0%	Theft, fraud, burglary, snatch theft, robbery, attack, sexual assault	Mental health (not otherwise specified)
18	Tripathi (2019)	India	Qualitative	6	Not reported	0%	0%	Cyber-crime	Qualitative impact
19	Bailey (2020)	UK	Qualitative	80	Not reported	71%	Not reported	Scams	Qualitative impact
20	Muhammad (2021)	India	Cross-sectional	402	Not reported for sub-sample	Not reported for victim sub-sample	Not reported but conducted in India	Violent crime (assault, mugging, threat to life or others)	Major depression

PTSD = post-traumatic stress disorder; PC-PTSD = The Primary Care PTSD Screen; ICD = International Classification of Diseases; RCT = Randomized Controlled Trial; LGB = lesbian, gay and bisexual; N/A = Not applicable; RCT = Randomized Controlled Trial.

### Quality Appraisal

Quality appraisal using the MMAT (Hong et al., 2018) was conducted for the 20 studies, an overview of which is presented in the supplementary material. Studies of low methodological quality were not excluded as we aimed to appraise the strengths and limitations.

Observations across the literature included: (a) Many studies lack detail on methods. (b) It was often unclear which crimes were included in studies. (c) Reporting of sample characteristics (including mean age, gender, and ethnicity) was inconsistent. (d) Of the studies that did report ethnicity, samples were predominantly white. (e) Studies varied on how long after the crime the outcome was measured; in some cases, the crime may have occurred much earlier in life. (f) Different definitions of psychological impact made comparisons across studies challenging. (g) Four studies included older victims alongside other populations or traumas, meaning examination of psychological impact in older crime victims was brief. (h) There was little consideration of how differences in frailty, physical health, and global functioning may influence outcomes. (i) Stranger-perpetrated and known-perpetrator crimes were often not clearly distinguished. (j) No studies obtained data on pre-crime psychological health limiting inferences around causality and direction thereof. (k) Studies often did not declare whether ethical approval was obtained or outline ethical considerations in their research. (l) Only feasibility intervention data has been published. (m) The challenges of timely identification and recruitment of older victims meant psychological impact studies were often based on convenience samples or randomly selected samples with low response rates, risking bias, and limiting generalization. Intervention studies addressed this by collaborating with police or healthcare agencies, although this limited samples to those known to those services.

### Narrative Synthesis

Our synthesis is divided into psychological impact and psychological intervention findings, with psychological impact studies further organized by study aims. The psychological impacts we identified across studies are summarized in Table 2, psychological impact methods and data are in Table 3, psychological intervention methods and data are in Table 4, and implications of this review for practice, policy, and future research are in Table 5.

**Table 2.** List of Psychological Impacts Identified.

1. Upset	7. Poor sleep	13. Post-traumatic stress disorder	19. Depression	25. Flashbacks
2. Frightened	8. Intrusive thoughts	14. Fear of further crime	20. Reduced self-esteem	26. Stress
3. Disbelief	9. Feeling vulnerable	15. Fear of going out/agoraphobia	21. Loss of trust/mistrust	27. Panic attacks
4. Crying	10. Insecurity	16. Fear of staying alone	22. Loss of confidence	28. Self-blame
5. Embarrassment	11. Anger	17. Impaired concentration	23. Shame	29. Shock
6. Fatigue	12. Annoyance	18. Anxiety	24. Skepticism	30. Behavior changes
Behavioral changes				
1. Avoidance of social activities	2. Carrying as little money as possible	3. Locking doors and windows	4. Avoidance of online banking	5. Monitoring belongings in crowded places

**Table 3.** Summary Findings of Studies Reporting on Psychological Impact.

First Author	Setting	Study Aim	Recruitment Procedure	Length of Time Since the Crime	Time Between Assessment and Follow-up	Crime Assessment	Outcome Measures	Data Analysis	Results	Strengths	Weaknesses
Krause (1986)	Community	To examine whether social support buffers the deleterious effects of crime on depressive symptoms	Random household selection	Events occurring within the previous year	Not applicable	Stressful Life Events Questionnaire (created for the study)	CES-D (Radloff, 1977)	Ordinary least squares multiple regression	Post-crime depressive symptoms were lower in older victims with high emotional support (*b* *=* 0.689) than those with low emotional support (*b* *=* 1.17). Minimal differences were found in post-crime depressive symptoms between older adults with high informational support (*b* = 0.641) compared to low informational support (*b* *=* 0.630) (*N* = 332).	Considers explanatory factors and protective factors; large sample size; asked about recent crimes; standardized measure of depression	Recall bias; crime assessed as an umbrella category including legal disputes
Jones (1987)	Sheltered housing	To describe impact of crime on victims	Data from complaints to wardens	Not reported	Not applicable	Complaints to wardens	Not used	Not used	Described older victims of burglary and distraction burglary as distressed and upset. Common feature was that all happened inside their homes.	Early attempt to understand impact in older victims and explanatory factors	Cannot be generalized, did not consider crimes outside of housing association, limited to complaint data, no standardized measures
O’Niell (1989)	Hospital in-patients	To compare burglary impact between older and younger people	Admissions to hospital	2 years	Not applicable	Self-report	Not reported	χ^2^	36% reported anxiety or depression.	First of its kind, clinical recommendations made	Small sample, one study site, no standardized measures, not generalizable
Norris (1992)	Community	To determine impact of different trauma events (including crime) on different demo-graphic groups (including older adults)	Investigator toured an urban area for signs of hurricane damage and selected areas with similar demo-graphics	Lifetime and past year	Not applicable	Traumatic Stress Scale (Norris, 1990)	Clinical interview to assess DSM-III-R PTSD; Perceived Stress Scale (Cohen et al., 1983)	ANOVA	Older persons showed consistently lower rates of PTSD with regards to crime compared to younger people (statistics unreported).	Included past year assessment.	Only limited attention given to crime; high risk of selection bias; high refusal rate;
Tyra (1996)	Community	Case study description of an older rape victim reported by a nurse	Older victim referred to nurse for treatment	Case summary of acute and longer term but exact time scales not reported	Not reported	Clinical summary	Not used	Qualitative	Acute rape trauma syndrome: disbelief, crying, embarrassment, sleep difficulties, intrusive thoughts, sense of vulnerability, anger. Long-term: fear of going out, fear of staying alone at night, inability to concentrate at work, intense anger, behavior changes (doors/windows locked, curtains closed)	Detailed, considered changes over time, included follow-up, considered internalizing and externalizing emotions, recommendations for interventions	Cannot be generalized, standardized measures not used, rape trauma syndrome not included in current DSM or ICD.
Simpson (1996)	Old age psychiatry community clinics	Assessment of impact of crime + 5 case studies on impact in older victims	Convenience sample	Not reported	Not applicable	Self-report	Checklist for DSM-IV PTSD and depression, Mini Mental State Examination; Impact of Events Scale (Sundin & Horowitz, 2002)	Descriptive	100/ 350 (28.5%) older adults had been victims, of which 5 (5%) had PTSD. Clinical characteristics included depression, insomnia, avoidance, agoraphobia.	Amongst the first to provide data on PTSD in older crime victims in the UK and highlighted need for epidemiological studies; gave recommendations for clinicians	Conducted in one health center so cannot be generalized; lack of detail in methodology limiting replicability; limited demographic details reported; limited to older adults known to psychiatry services; anxiety not assessed
Spalek (1999)	Community	To explore the impact of financial crime in older victims of the Maxwell Pension Scandal	Purposive sampling of support groups	Not clearly reported, however, scandal was discovered in 1996 and paper published in 1999	Not applicable	Self-reported victim	N/A	Qualitative, no further details reported	Themes reported were anger, anxiety, shattered assumptions (Janoff-Bulman, 1985). Anger noted in place of self-blame.	First to consider fraud, in-depth exploration of a specific incident	Limited reporting of qualitative methods including whether one-to-one or focus groups, approach to analysis, reflexive/epistemological assumptions. Deductive analytic approach means unanticipated themes may have been missed.
Acierno (2004)	Community	Preliminary evaluation of a video-based intervention for older adult victims of violence	Through contact with LEVA	Contacted attempted within 2–3 weeks of the crime	6 weeks	Crime reported to LEVA	Psychotherapeutic and safety planning knowledge quiz, Geriatric depression scale (Scogin et al., 2000), PTSD symptom scale (Foa et al., 2016), Beck Anxiety Inventory (Beck & Steer, 1990)	Not applicable—No data on psychological distress pre-treatment collected	Not applicable—No data on psychological distress pre-treatment collected.	Evaluated in intervention table as no pre-treatment data collected	Evaluated in intervention table as no pre-treatment data collected
Brunet (2013)	Hospital	Compare outcomes in older victims of assault and motor vehicle accidents	Access to A&E	Peri-traumatic distress assessed within seven days of the event	PTSD symptoms assessed at 1, 6 and 12 months after the event	Seeking medical treatment for assault	Peritraumatic Distress Inventory, Peritraumatic Dissociative Experiences Questionnaire, Clinician-administered PTSD scale	Generalized linear mixed models	Severity of PTSD in older adults is greater after violent assault than motor vehicle accidents; peritraumatic distress is a risk factor for PTSD.	Systematic approach to recruitment and follow-up; investigates possible explanatory pathways to mental health outcomes; approach to missing data reported	Small sample size, low acceptance rate in participants (29%), did not study anxiety or depression
Fredriksen-Goldsen et al. (2014)	Community	Mental health risk factors in transgender older adults	Surveys	Lifetime	Not applicable	Lifetime Victimization Scale (D’ Augelli & Grossman, 2001)	Centre for Epidemiological Studies Depression Scale (Radloff, 1977)	Linear and logistic regression	Transgender older adults reported higher rates of lifetime victimization than non-transgender lesbian, gay and bisexual older adults; victimization and stigma explained the highest proportion of the total effect of gender identity on health outcomes adults	Large sample, understudied population; measure validated for older adults	Recruitment limited to mailing lists, mostly urban areas, assessment of crime brief
McGraw (2006)	Community	Report findings from a nurse-link scheme	Police referral	Nurse visit joint with police as part of initial response to the crime report	3 months	Police reported distraction burglary	Geriatric Depression Scale (Scogin et al., 2000), Hospital Anxiety and Depression Scale (Zigmond & Snaith, 1983)	Descriptive statistics	Depression was reported by 25% and anxiety was reported by 13% during first visit.	Collaborated with police, used standard measures, set time between crime and assessment.	Follow-up data on psychological outcomes not reported, not a randomized controlled trial.
Cross (2015)	Media release invite from police to known victim	Qualitatively examine experiences in older victims of fraud or attempted fraud	Face-to-face semi-structured interviews	Not reported	N/A	Self-reported receipt of fraudulent email	None	Thematic coding	Victims experienced self-blame, shame, embarrassment, and endorsed victim-blaming myths. Humor was an ineffective coping mechanism serving as a barrier to disclosure.	Explored impact of attempted fraud as well as substantive fraud.	Limited reporting of qualitative methods or analysis, no reflexivity statement
Iganski and Lagou(2015)	Community	Secondary analysis on crime survey data to understand who is most affected by racially-motivated hate crime.	Secondary analysis of Crime Survey for England Wales (CSEW)	Previous 12 months	Not applicable	Data from CSEW	Asked to tick which emotions they had felt	Logistic regression	Older adults at increased risk of reporting internalized emotions but no/minimal increased risk of externalized emotions to racially motivated hate crime, results are non-significant for both. Odds are lower than younger age groups.	Specific analysis of hate crime, within a set time-frame, the CSEW includes victims that did not report to the police, considers internalized and externalized emotions.	Limited detail on older adults, self-report outcome, not assessed for validity or reliability; possibility of under-reporting not considered; large confidence intervals.
Tan and Haining (2016)	Community	To explore how crime affects health and quality of life in different demographic groups	Postal questionnaire	Previous 5 years	Not applicable	Not reported	Not reported	Descriptive statistics	Respondents aged 61–75 reported: stress (20%), sleeping difficulties (10.2%), lack of confidence (14.8%), depression (8%), panic attacks (9.1%) In respondents aged 75+: stress (13.5%), lack of confidence (11.5%), depression (7.7%), panic attacks (7.7%).	Breakdown of older adults into two age groups; good sample size	Low response rate (20%); crime and symptom assessment unclear; studied alongside other demographic groups so limited exploration
Serfaty (2016)	Community	To identify mental health problems in older victims of common crime, provide preliminary data on prevalence, and test feasibility of RCT	Older victims, identified through police teams, were screened for symptoms of anxiety, depression, or PTSD one and three months after a crime	Within the previous month	3 months	A crime reported to the police	Kessler-6 (Kessler et al., 2003); PC-PTSD (Prins et al., 2004); GAD-2 (Kroenke et al., 2007); PHQ-2 (Kroenke et al., 2003)	χ^2^ comparison of crime type by caseness at 3 months	Of those who were re-screened, 27.6% were cases on one of the measures. Of these, 88/134 agreed to diagnostic assessment of which 33/80 (40%) met diagnostic criteria after the crime.	Tested different ways to identify and recruit older victims, conducted intervention feasibility work, standardized screening tools used	Sample not representative of the general population; small sample size as pilot study
Reisig (2017)	Community	Investigating familial ties and behavior avoidance on depressive symptoms after victimization	Survey data collected through random digit dialing	Crime over the past year	Not applicable	Crime (and family ties) assessed through questionnaire designed for the study	Geriatric Depression Scale (Scogin et al., 2000); avoidance, family ties and crime assessed through questionnaires designed for the study	Linear regression	Victims reported higher depressive symptoms and greater behavioral avoidance coping. The link was weaker among participants with strong attachments to their spouse and adult children Criminal victimization was significantly associated with avoidance coping and strong spousal attachment was negatively associated with avoidance coping.	Breakdown of age groups within older victims; followed on from existing research	Avoidance coping not assessed through a validated measure, does not consider physical health as confounder of avoidance
Qin (2018)	Community	Mental health after a crime in older adults in China	Multi-stage sampling	Lifetime	Not applicable	Seven item questionnaire developed for the study	The Fear of Crime scale (Ferraro & LGrange, 1987); The 12-item General Health Questionnaire (Goldberg, 1972)	Hierarchical regression	254 (56%) reported one or more types of common crime and experience of common crime was significantly correlated. with poorer mental health and constrained behavior. When controlling for sociodemographic characteristics and physical health, common crime victimization was consistently a significant predictor of poor mental health (β = .126, *p* = .006).	First non-western study, controlled for sociodemographic features and health, analyzed stranger-perpetrated and domestic violence crimes separately	Low response rate (57%); mental health symptoms treated as a homogenous concept; not broken down into specific disorders or symptoms
Tripathi (2019)	Community	Qualitatively explore older male’s experiences of cybercrime	Through professionals also interviewed who known through local contacts.	Not reported	Not applicable	Identified through professionals known to the interviewer	Not a	Theoretical thematic framework approach	The crime led to persistent and unresolved feelings of shame, depression and anxiety.	Impact in non-western country.	Limited details reported; brief discussion on impact
Bailey (2020)	Community	To report data from 80 older adults’ written responses to a Mass Observation Archive Directive focused on scams	Written responses captured through the Mass Observation Project	Lifetime	Not applicable	Asked to write free-text response to: “Have you ever been a victim of scam? What happened?”	Asked to write free-text response to: “how did it affect your mental health?”	Thematic	Identified anxiety, victim blaming, and protective behavior including skepticism, avoiding cold callers, screening contacts/email.	Cost-effective approach to data collection, written responses facilitate anonymity reducing socially desirable responding	Limited details of qualitative methods, no reflexivity statement, written responses only; overly white British sample, non-response rates not recorded
Muhammad (2021)	Community	To investigate the association of crime victimhood with depression	Data drawn from the Longitudinal Ageing Study in India Wave 1 2017–2018, which used multi-stage stratified area probability cluster sampling	Previous 12 months	Not applicable	In the last 12 months, have you been a victim of a violent crime such as assault/ mugging/ threat to life/ others? (Yes/No)	CIDI-SF (Gigantesco & Morosini, 2008)	χ^2^ and logistic regression	17.70% of older adults who were victims of a violent crime were suffering from depression against 8.52% non-victims. Older victims of violent crime had higher odds of suffering depression compared to non-victims. Once socio-economic and health variables were adjusted for, crime victims were 84% more likely to be depressed than non-victims. Older female victims of violent crime had 2.6 odds of suffering depression. Older victims of violent crimes residing in rural areas had higher odds of suffering depression.	Large scale nationally representative sample in India conducted in every state/union territory, considered risk factors, used standardized measurement tool for depression	Cannot establish causality, self-reported data on crime, sample characteristics for victim sub-set not reported

*Note*. LEVA = Law Enforcement Victim Advocates; CIDI-SF = Short Form Composite International Diagnostic Interview; PHQ = Patient Health Questionnaire; GAD = General Anxiety Disorder; ANOVA = analysis of variance; CES-D = Centre for Epidemiologic Studies Depression Scale, PC-PTSD = The Primary Care PTSD Screen..

* *p* ≤ .05. **p ≤ .01.

**Table 4.** Summary Findings of Studies Reporting Interventions.

First Author (Year)	Country	Study Design	Sample Size (*N*)	Participants				Intervention	Comparison	Outcome	Findings	Strengths	Limitations
				Crime	Age (Mean)	Gender	Ethnicity						
Tyra (1996)	USA	Case study	1	Rape	64 (N/A)	Female	Not reported	Crisis counselling Acute: (psycho-education, empathetic listening, reassurance) Long-term: (supporting healthy coping strategies, linking into social support and psycho-education on emotions during court hearing)	Not applicable	Description	Victim maintained independent living. A year later she reported that flashbacks continued and that she will never feel safe again.	First intervention, holistic, considers acute and long-term coping, example of delivery alongside healthcare	Evaluation not possible as descriptive case study
Acierno et al. (2004)	USA	RCT	116	Violence	55 + (66.9)	66.5% female	53% Caucasian	Psycho-education video on healthy coping and safety planning	Existing advocacy services	GDI, BAI	Those who received the video had greater knowledge that day but no difference in depression or anxiety scores were found at 6-week follow-up.	Cost-effective intervention; partnership with local services to identify older victims of recent crimes; use of standardized measures	Genuine effects may have been missed as i) no power calculation, ii) no baseline measure, iii) participants selected on victim status rather than distress status and iv) delivery of intervention within two-three weeks
McGraw (2006)	UK	Descriptive	77	Distraction burglary	70+ (79)	Not reported	Not reported	Outreach from district nurses to address health and social care needs	None	GDI, HADS	Identified depression in 25% and anxiety in 13%. Positive examples of support discussed.	Outreach provided through existing services; mental health measured used standardized measures	Descriptive study so evaluation not possible
Serfaty et al. (2016)	UK	RCT	26	Property, fraud, violent crimes	55 (71.7)	55.2% female	86.1% Caucasian	Cognitive-behavioral therapy informed victim improvement package	Treatment as usual	WHODAS-II; BDI-II; BAI; PDS	Favorable trends for treating anxiety and depression but not PTSD.	Partnered with services; acceptability considered; randomization procedure reported; researchers blinded	Feasibility study preventing full evaluation; no talking control

*Note*. BAI = Beck Anxiety Inventory; BDI-II = Beck Depression Inventory II; PDS = Post-Traumatic Stress Diagnostic Scale; GDI = Geriatric Depression Inventory; HADS = Hospital Anxiety Disorder Scale; WHODAS-II = World Health Organisation Disability Assessment Schedule.

**Table 5.** Implications of This Review for Practice, Policy, and Future Research.

Practice and policy	1. As adverse psychological consequences are consistently reported in older crime victims, health-care professionals should consider whether intervention is needed to reduce chronicity of symptoms. The police are advised to signpost distressed older victims to their GP; however, further research is needed to establish whether this is sufficient for service uptake in this population, or whether assertive outreach is warranted.
	2. Findings that PTSD symptoms are greater in older victims of violence than older victims of motor vehicle accidents (Brunet et al., 2013), and that depressive symptoms have a potentially bigger health impact than well-established risk factors including smoking and obesity (Fredricksen-Goldsen et al., 2014), support growing calls to view the psychological impact of crime on victims as a public health concern (Burnes et al., 2017; Tan & Haining, 2016).
Future research	1. Fully-powered trials testing the effectiveness of psychological interventions for distress in older crime victims are needed. Researchers are advised to establish partnerships with services in addition to the police, such as faith organizations, to aid timely identification of older victims who do not report their crime and to reduce selection bias.
	2. Inductive qualitative studies are needed to understand whether there are any further psychological impacts.
	3. Studies to establish prevalence rates in representative samples are needed.
	4. Further research on risk factors is needed to understand which older victims are adversely affected and most in need of support.
	5. Studies to clarify how different victim-perpetrator relationships influence psychological outcomes in older victims.
	6. Studies should be carefully planned to facilitate inclusion of under-served sub-groups of older victims.
	7. Establishing the extent community crime affects older people in residential care and the psychological impact of this.
	8. Studies to clarify differences in short-term and long-term psychological impact of crime in older victims.

### Psychological Impact

Through synthesis of all study data, we identified 30 different psychological impacts, outlined in Table 2.

### Case Studies

Two early studies from the United Kingdom and United States provided case study data (Jones, 1987; Tyra, 1996). The UK study summarized twelve crime-related incident reports from wardens of sheltered accommodation, which described older victims of burglary and vandalism as “*upset*” and “*frightened*” (Jones, 1987). Fraudulent entry and theft was reported to “*affect them profoundly*,” and it was suggested this may be because of emotional attachment to their home (Jones, 1987, pp. 195–96). The U.S. study described acute and chronic experiences of “rape trauma syndrome” in an older rape victim (Tyra, 1996). Acute experiences included disbelief, recurrent crying, embarrassment, fatigue, poor sleep, intrusive thoughts, feeling vulnerable, insecurity, and intense anger. Chronic experiences included fear of going out at night or staying alone, poor concentration, intrusive thoughts, and ongoing anger. Together, these studies provide insight into the nature of psychological impact in older victims but cannot be generalized due to insufficient numbers.

### Qualitative Studies

Four qualitative studies were identified, all focusing on fraud, indicating an evidence gap for qualitative research in older victims of other crimes. These included older victims of the Robert Maxwell Pension scandal in the United Kingdom (Spalek, 1999), attempted and completed scams in Australia and the United Kingdom (Bailey et al., 2020; Cross, 2015), and cyber-scams in India (Tripathi et al., 2019). Reported themes on psychological impact in these studies included: anxiety, depression, reduced self-esteem, loss of trust and confidence, embarrassment, shame, increased skepticism, mistrust, and behavior changes (e.g., avoiding online banking). Self-blame was observed in older victims who felt personally responsible but was absent in older victims scammed by a trusted pension provider; anger was noted instead (Spalek, 1999). It is a strength that two studies considered both attempted and completed scams, but contradictory findings were observed: one observed psychological distress in victims who suffered severe financial losses only (Cross, 2015), the other reported that malicious intention was more distressing (Bailey et al., 2020).

Notable across all four studies was limited detail on interviewing approach (e.g., one-to-one, focus groups), topic guide (e.g., semi-structured), analytical approach, theoretical assumptions, and reflections on researcher background and possible personal biases. All four appeared to approach the data deductively, seeking evidence to support existing ideas on psychological impact, but did not inductively explore whether there were other impacts not yet considered. Taken together, these studies support that fraud adversely affects older victims but inductive analysis with older victims of different crime types is needed to understand the full range of impact.

### Descriptive Surveys

Three studies conducted surveys in larger samples of older people asking whether they had been a victim previously and, if so, what psychological impacts they had experienced. The first was in older hospital inpatients in Ireland (*N* *=* 272) (O’Neill et al., 1989), which found that of those who reported having been burgled in the previous two years (*n* *=* 72, 26%), almost all (90%) reported a psychological impact. These included fear of further crime (*n* *=* 57, 79%), anxiety or depression (*n* *=* 36, 50%), sleep disturbance (*n* *=* 32, 44%), and agoraphobia (*n* *=* 32, 44%). The second study was in older adults accessing a community mental health clinic in Manchester, United Kingdom (*N* *=* 350) (Simpson et al., 1996). This study found that 100 participants reported having been crime victims; of whom, five (5%) met diagnostic criteria for PTSD. It was not reported when the crimes occurred nor what the clinical presentation of the 95 victims who did not meet diagnostic criteria for PTSD was like, limiting comparisons between these two studies. These two surveys were also conducted in convenience samples of older people accessing healthcare so cannot be generalized to all older victims.

The third study tried multistage sampling 800 older people living in urban China; however, the response rate was low (*n* *=* 453, 57%) (Qin & Yan, 2018). Over half of respondents had experienced one or more types of crime (*n* *=* 254, 56%). Theft (*n* = 198, 44%), fraud (*n* = 140, 31%), and burglary (*n* = 253, 56%) were most frequently reported while physical assault (*n* = 5, 1%) and rape (*n* = 1, 0.2%) were least reported. Experience of crime was a consistently significant predictor of poor mental health on the General Health Questionnaire (Pan & Goldberg, 1990), even when physical health and sociodemographic characteristics were controlled for.

Taken together, these three studies suggest high levels of distress is associated with being a victim of crime, although they cannot be generalized to all older victims. As psychological health before the crime was not known, it is unclear whether poor mental health was because of the crime or preexisting.

### Psychological Impact in Older Victims Compared to Older Non-Victims

One recent study of depression using data from a large national survey on ageing in India (*N* = 31,646) showed that 1.32% of respondents (*n* *=* 402) reported having been victims of violent crime, such as assault or mugging, in the previous 12 months (Muhammad et al., 2021). Using the Short-Form Composite International Diagnostic Interview for major depression, 17.7% scored positive compared to 8.52% of non-victims (U﻿nadjusted odds ratio (UOR): 1.54, 95% CI [1.05–2.26]). After controlling for sociodemographic and health variables, the adjusted odds ratio increased to 1.84 (95% CI [1.15–2.95]). A strength of this study is that it reports a representative sample and it sought to measure the association between crime and depression within a defined time-period. However, the direction of association remains unclear, and findings may not be generalizable to nonviolent crime.

### Psychological Impact of Crime Compared to Other Trauma Types in Older Victims

One study compared psychological outcomes after crime with another trauma-type in older victims (Brunet et al., 2013). Older adults seeking emergency medical treatment for either physical assault or motor vehicle accidents were assessed for peritraumatic distress symptoms, and followed up 1 week and 1, 6, and 12 months post-incident. Older victims of physical assault scored significantly higher across time-points on the Clinician Administered PTSD Scale (CASP) (mean = 36.1, 95% CI [22.8–49.3]) than older victims of motor vehicle accidents (mean = 15.2, 95% CI [10.8–19.7]) (*t* = 2.23; *p* *=* .03). Consistent with the ageing survey in India, this demonstrates severity of psychological impact in older victims of violence. By using the gold-standard assessment tool for PTSD (CASP) and partnering with a local hospital, assessment was at clearly defined time-points. However, research to understand the severity of psychological impact in older victims of less violent crimes is also needed.

### Older Victims Compared to Younger Victims

Three studies investigated psychological distress across age groups. One found no difference between younger and older victims (Tan & Haining, 2016) and two found lower symptoms in older adults than younger adults (Iganski & Lagou, 2015; Norris, 1992). However, these retrospective studies asked participants whether they had been a victim anytime in their lives, meaning acute distress was not measured in older adults and responses may have been affected by recall biases. Results may also have been conflated in these studies. For example, one study (Tan & Haining, 2016) found that female victims of all ages were more likely to report psychological symptoms than males but did not provide a gender breakdown within age groups; lower distress scores in older males may have skewed higher distress scores in older females. Researchers should consider whether there is clinical value in investigating whether one age group is more affected than another as erroneous conclusions in either direction risks excluding vulnerable populations from research.

### Risk Factors for Adverse Psychological Outcomes in Older Victims

While studies often analyzed older victims as one group, some have begun to consider the factors that may contribute to distress within this population.

### Type of Crime

Psychological distress was reported in older victims of assault (Acierno et al., 2004; Brunet et al., 2013), rape (Tyra, 1996), fraud (Bailey et al., 2020; Cross, 2015; Spalek, 1999; Tripathi et al., 2019), burglary (Jones, 1987), distraction burglary (McGraw & Drennan, 2006), mugging (Muhammad et al., 2021), and vandalism (Jones, 1987), but there is less on the impact of low-value high-frequency crimes such as petty theft. It therefore remains unclear whether psychological distress arises from all crime or specific crime types. Equivocal findings in the fraud studies (Bailey et al., 2020; Cross, 2015) also raise the question whether it is the crime itself or the malicious intent that is distressing in older victims.

### Individual Characteristics

Comparatively few studies reported on the influence of individual differences on psychological outcomes. The surveys of older people in urban China (Qin & Yan, 2018) and across India (Muhammad et al., 2021) both found a strong association between experiencing a crime and mental health even after variables such as health, gender, age, education, household finances, and living arrangements were controlled for. Of the victims of violent crime in India, older females living in rural areas had the highest odds of suffering major depression (Adjusted odds ratio (AOR): 2.27, 95% CI [1.25–4.14]) (Muhammad et al., 2021). Further research is needed on the influences of sociodemographic and health variables on psychological outcomes across different crime types.

Two other studies highlight that crimes targeted toward marginalized groups (called “hate crime” in the United Kingdom and “bias crime” in the United States) may be particularly distressing. A study in the United States (Fredriksen-Goldsen et al., 2014) found that transgender older adults (*n* *=* 174) reported higher lifetime victimization and depressive symptoms than lesbian, gay, and bisexual (*n* *=* 2,201) older adults. Victimization and stigma explained the highest proportion of the total effect of gender identity on health outcomes in transgender older adults, even when compared to other health-related behaviors including smoking and obesity. Another study examining data from the Crime Survey for England and Wales found older victims of racially motivated hate crime were at increased risk of self-reported internalized emotions, *although standardized outcomes measures were not used and the findings were not statistically significant* (Iganski & Lagou, 2015).

### Social Isolation

Two studies suggest that older victims with limited social support may be at risk. *An early study* (*N* = 332) found that depressive symptoms were lower in older crime victims with high emotional support than those with low emotional support, although this was not statistically significant and minimal differences were found between those with high informational support and low informational support (Krause, 1986). However, these findings were supported by a more recent study (*N* *=* 2,000) which found that although older victims reported increased depressive symptoms after crime, the link was significantly weaker in those with strong attachments to spouse and adult children (Reisig et al., 2017).

### Changes in Behavior

Several studies reported behavior changes in older victims including avoiding online banking, avoiding unidentified callers, and increasing home security (Bailey et al., 2020; Qin & Yan, 2018; Tripathi et al., 2019). Crime victimization was found to significantly correlate with “constrained behaviors” in older people in China (Qin & Yan, 2018) and avoidant behaviors in older people in the United States (Reisig et al., 2017). Strong spousal attachments were also found to moderate this link (Reisig et al., 2017). However, while these studies tested a relationship between crime and protective behaviors, and crime and mental health, neither tested a relationship between protective behaviors and mental health. Protective behaviors were also measured based on presence or absence (Qin & Yan, 2018) or frequency (Reisig et al., 2017), which may not be valid in older victims as they were not asked whether this was a change since the crime. In the survey in China, nearly all older people (98.5%) reported engaging in protective behaviors yet only 56% reported having been victims, making it unclear whether these behaviors were in response to the crime or preexisting. The American study (Reisig et al., 2017) also defined avoidance as nonattendance at activities such as the cinema or leisure sports, which may have been confounded by poor physical health. Nonetheless, given several studies observed behavior changes in older victims, further investigation using valid assessment tools to strengthen understanding of these associations is needed.

Taken together, these findings suggest that crime type, sociodemographic characteristics, social isolation, and behavior changes are possible factors that may influence how older victims psychologically respond to a crime.

### Interventions

Four feasibility intervention studies for psychological distress in older victims were identified: two nursing schemes (McGraw & Drennan, 2006; Tyra, 1996), a psychoeducation video (Acierno et al., 2004), and an adaptation of cognitive-behavioral therapy (CBT) (Serfaty et al., 2016). There are no published data from fully-powered trials, although further testing of the adapted cognitive-behavioral therapy is in progress (Serfaty et al., 2020).

The first nursing intervention was crisis counselling for an older rape victim (*N* *=* 1) delivered alongside medical care (Tyra, 1996). This involved psychoeducation and empathetic listening in the aftermath, longer term linking into social support, and psychoeducation on coping during court proceedings. It was reported that whilst the older victim continued to suffer flashbacks and felt unsafe a year later, crisis counselling meant she was able to continue living independently at home. Standardized outcome measures were not used and, as a case study, the findings cannot be generalized. The aftermath period that crisis counselling was delivered in was not defined, but it is important to note that guidance released since cautions against prematurely intervening before the person has had a chance to recover naturally (NICE, 2018).

The second study described a “nurse-link” collaboration between district nurses (also called “home visiting” nurses) and London police to assess and address health and social care needs of distraction burglary victims aged 70 and over (*N* = 77) (McGraw & Drennan, 2006). Of these, 19 (25%) scored positive for depression on the Geriatric Depression Scale and 10 (13%) on the Hospital Anxiety and Depression Scale. Health and social care were coordinated for these older victims and follow-up case studies describe successful outcomes. While it is not possible to evaluate the effectiveness of descriptive studies, further feasibility research would be worthwhile as this highlights the possible benefits that could be achieved by embedding support within existing nursing services.

The psychoeducation video was delivered in “real-world settings” by local police for older victims of violence in South Carolina (*N* *=* 116) and was compared to existing advocacy in a pilot randomized controlled trial (Acierno et al., 2004). Participants who received the video had greater knowledge when assessed later that day, but no differences were found between treatment groups on the Geriatric Depression Inventory or Beck Anxiety Inventory at 6-week follow-up. Genuine differences may have been missed as power calculations were unreported, inclusion was based on victim status rather than distress scores, the intervention was delivered within a few weeks of the crime, researchers were not blinded, randomization technique was not reported, and missing data was high (29%). Baseline assessment was not conducted so there was no data on pretreatment psychological distress scores, and knowledge retention at follow-up was not collected so within-groups changes could not be assessed. Further feasibility work addressing these limitations is recommended as a psychoeducation video would be low cost to deliver if found clinically effective.

Serfaty et al. (2016) partnered with local police and Victim Support to systematically identify older victims of different crime types within a month of reporting a crime, and screen for depression, anxiety, and PTSD at 1 and 3 months after. As many as 27.6% of older victims were found to have continued distress symptoms 3 months later, but this small feasibility sample was not representative of the general population, providing only a preliminary prevalence estimation. Those who continued to screen positive 3 months post crime were offered a diagnostic interview. Of those, *N* *=* 26 participated in a feasibility RCT of modified CBT compared to treatment-as-usual. Modified CBT was found to be acceptable and appeared promising for treating anxiety and depression but not PTSD. It is a strength that this study systematically identified, assessed, and treated older victims using standardized measures within a defined timeframe. Further evaluation through a fully powered RCT is needed to determine the efficacy of this treatment, which is now underway (Serfaty et al., 2020).

## Discussion

This is the first systematic narrative review and quality appraisal of existing research on psychological impact and interventions for older crime victims living in the community. Through systematic searching we found 20 studies on psychological distress in older victims, 4 of which included interventions.

Our first aim was to synthesize existing evidence on psychological impact in older victims. While studies varied across aims, outcome definitions, and included crimes, adverse impacts in older victims were consistently reported. Thirty different psychological impacts were identified, with particular emphasis on internalized anxious and depressive symptoms. Initial prevalence estimates that 28% of older victims of different crime types continue to suffer depression and/or anxiety 3 months later (Serfaty et al., 2016) is considerably higher than rates for depression (7%) and anxiety (4%) in older people globally (WHO, 2017). For violent crime, depressive symptoms are even higher (Muhammad et al., 2021) and greater PTSD symptoms have been reported than in older victims of motor vehicle accidents (Brunet et al., 2013). Depression attributed to victimization has a potentially bigger health impact than well-established risks including smoking and obesity (Fredriksen-Goldsen et al., 2014). Our findings follow on from earlier physical health studies which found older victims of violence and burglary are at increased risk of nursing home placement and accelerated mortality (Donaldson, 2003; Lachs et al., 2006). Together, this supports growing calls to acknowledge the impact of crime on victims as a public health concern (Burnes et al., 2017; Tan & Haining, 2016).

Our second aim was to identify existing interventions for psychological distress in older victims. While intervention research is in its infancy, it is encouraging that modified CBT was acceptable and favorable for treating anxious and depressive symptoms (Serfaty et al., 2016) and that this is now being further tested (Serfaty et al., 2020). Partnering with local healthcare and police services also appears promising for timely identification, recruitment, and intervention delivery (Acierno et al., 2004; McGraw & Drennan, 2006). As not all crimes are reported, and uptake of mental health services is low in both older adults and crime victims of all ages (MacDonald, 2002; McCart et al., 2010; Nair et al., 2019), future research should consider other partnerships to engage older victims not known to these services. This may include faith organizations, as promising results have been found for spiritually-focused group interventions in older female victims of domestic violence (Bowland et al., 2012). Interventions targeted toward social inclusion and enhancing quality of social connections may also be beneficial (Zagic et al., 2021) given findings that social support moderates distress in older victims (Reisig et al., 2017). While randomized controlled trials are the gold-standard for intervention research, single case experimental design may be more cost-effective for testing interventions in older crime victims as statistical power can be achieved through repeat-testing fewer participants compared to conventional trials (Brysbaret, 2019).

Our third and final aim was to quality appraise the literature so that recommendations to strengthen the evidence can be made. Priorities for future research include establishing prevalence in representative samples, understanding how different crime types and victim-perpetrator relationships influence outcomes, understanding both the short-term and long-term impact of crime, and further feasibility and evaluation of interventions. Inductive qualitative research is needed to explore whether there are further impacts not yet identified. Few studies discussed externalized emotions such as anger, yet this was found to be an important part of the phenomenology of crime-related PTSD in violence victims aged 18 years and over (Andrews et al., 2000). Further research on risk factors is recommended as understanding which older victims are most adversely affected will ensure effective allocation of resources and development of targeted interventions. Studies should also be carefully planned to ensure accessibility to under-served groups (NIHR-INCLUDE, 2020). This may include older victims from ethnic minorities, who do not speak English, who lack trust in statutory services, are without permanent residency, or have mobility or sensory disabilities (Bodicoat et al., 2021).

Consistency of outcome definitions can be improved by adhering to DSM or ICD diagnostic criteria (American Psychiatric Association, 2013; WHO, 2004). Use of standardized assessment tools would strengthen study comparability. Validated tools include the Patient Health Questionnaire (Arolls et al., 2010) or Beck Depression Inventory (Beck et al., 1996) for depression, the General Anxiety Disorder (Plummer et al., 2015) or Beck Anxiety Inventory (Beck & Steer, 1990) for anxiety, and the Clinician Administered PTSD scale (Weathers et al., 2015) for PTSD. Use of the Traumatic Events Scale is also advised as it can discriminate between people with severe and mild distress to detect which individuals would most benefit from treatment (Sundin & Horowitz, 2002). Standardizing assessment and intervention to 3 months post-incident has been recommended in other trauma populations as it allows time for natural recovery without prolonging suffering in victims with continued symptoms (McNally et al., 2003). This would also build on existing research identified in this review (Serfaty et al., 2016, 2020).

While reporting guidelines were not available when many of the studies were published, future research should follow these to reduce missing detail, including CONSORT for RCTs, STROBE for observational studies, and SRQR for qualitative studies (The Equator Network, 2022). We recommend that researchers also specify which crimes have been included, when the crimes occurred, and whether the victim had a preexisting relationship with the perpetrator or not. As older adults range from individuals in good health and living independently to those who are frail and nearing end of life, clarifying which phase of late adulthood the study findings are most relevant to will help inform treatment decisions (Jaul & Barron, 2021). Finally, studies should confirm research ethics approval has been obtained and outlined how procedures adhere to ethical research guidance for older trauma victims (McGuire, 2009; Seedat et al., 2004). This includes obtaining informed consent, ensuring confidentiality, and safeguarding procedures if a participant were to disclose ongoing abuse.

Our systematic review is a comprehensive synthesis of a limited evidence base. While many studies included have bias risk, recommendations to improve methodological quality have been made. Only studies published in English were included, however, few studies published in other languages were noted during screening meaning the number of studies missed is likely to have been small. We purposefully included only peer-reviewed studies as recommended for reviews with limited resources as non-published studies often have more methodological issues and introduce further bias rather than reduce it (Egger et al., 2003). Our included studies focused on older victims living in the community as this is where most of these crime types occur, however, understanding the extent of such crime and its impact in older people in nursing homes and long-term residential care may be an important area to include in future research.

To conclude, there is growing evidence that older adults are adversely psychologically impacted by crime. Further research following methodological recommendations outlined in this review is urgently needed to improve outcomes in this neglected population.
